# Cloning a novel reduced-height (*Rht*) gene *TaOSCA1.4* from a QTL in wheat

**DOI:** 10.3389/fpls.2024.1381243

**Published:** 2024-05-16

**Authors:** Guangde Lv, Xuemei Jin, Hui Wang, Yijun Wang, Qun Wu, Haimeng Wu, Fangshan Jiang, Yanming Ma, Yanrong An, Mingxia Zhang, Ying Guo, Sishen Li

**Affiliations:** ^1^ National Key Laboratory of Wheat Improvement, College of Agronomy, Shandong Agricultural University, Tai’an, China; ^2^ Tai’an Academy of Agricultural Science, Tai’an, China; ^3^ Rizhao Academy of Agricultural Science, Rizhao, China; ^4^ Zibo Academy of Agricultural Science, Zibo, China; ^5^ Xinjiang Academy of Agricultural Sciences, Urumqi, China

**Keywords:** wheat, plant height (PH), reduced-height (Rht) gene, gene cloning, RNA interference (RNAi), quantitative trait locus (QTL), nonselective hyperosmolality-gate calcium-permeable channel 1.4 (*TaOSCA1.4*)

## Abstract

Reducing plant height (PH) is one of the core contents of the “Green Revolution”, which began in the 1960s in wheat. A number of 27 reduced-height (*Rht*) genes have been identified and a great number of quantitative trait loci (QTLs) for PH have been mapped on all 21 chromosomes. Nonetheless, only several genes regulated PH have been cloned. In this study, we found the interval of QTL *QPh-1B* included an EST-SSR marker *swes1079*. According to the sequence of *swes1079*, we cloned the *TaOSCA1.4* gene. We developed a CAPS marker to analyze the variation across a natural population. The result showed that the PH was significantly different between the two haplotypes of *TaOSCA1.4–1B* under most of the 12 environments and the average values of irrigation and rainfed conditions. This result further demonstrated that *TaOSCA1.4* was associated with PH. Then, we validated the *TaOSCA1.4* via RNAi technology. The average PHs of the wild-type (WT), RNAi lines 1 (Ri-1) and 2 (Ri-2) were 94.6, 83.6 and 79.2 cm, respectively, with significant differences between the WT and Ri-1 and Ri-2. This result indicated that the *TaOSCA1.4* gene controls PH. *TaOSCA1.4* is a constitutively expressed gene and its protein localizes to the cell membrane. *TaOSCA1.4* gene is a member of the *OSCA* gene family, which regulates intracellular Ca^2+^ concentration. We hypothesized that knock down mutants of *TaOSCA1.4* gene reduced regulatory ability of Ca^2+^, thus reducing the PH. Furthermore, the cell lengths of the knock down mutants are not significantly different than that of WT. We speculate that *TaOSCA1.4* gene is not directly associated with gibberellin (GA), which should be a novel mechanism for a wheat *Rht* gene.

## 1 Introduction

Wheat (*Triticum aestivum* L.) is one of the most important food crops in the world. By the year 2050, the world population is expected to reach 9.3 billion, and the demand for bread wheat is consequently increasing (Dong et al., 2019). Moreover, wheat production faces many difficulties associated with climate, environment and cultivation. Plant height (PH) has a significant effect on plant morphogenesis and yield in the field. The application of dwarf genes in wheat breeding has enhanced the lodging resistance of wheat and greatly increased its yield (Hedden, 2003; Berry et al., 2007; Guedira et al., 2010).

Reducing PH is one of the core contents of the “Green Revolution”, which began in the 1960s in wheat (Peng et al., 1999; Velde et al., 2021). Many efforts have been made to identify genetic loci affecting PH, and 27 wheat reduced-height (*Rht*) genes have been discovered (Lu et al., 2015; Mo et al., 2018; Song et al., 2023a). The PH of wheat is a quantitatively inherited complex trait that is strongly influenced by interacting genetic and environmental factors. A great number of quantitative trait loci (QTLs) for PH have been mapped on all 21 chromosomes (Börner et al., 2002; Huang et al., 2003; Sourdille et al., 2003; Liu et al., 2011; Griffiffiffiths et al., 2012; Guan et al., 2018; Saini et al., 2022; Singh et al., 2022). Of these, some QTLs may be associated with *Rht* genes. Using ‘Jingdong 8/Aikang 58’ recombinant inbred lines (RILs), *QPh.caas-4D* was confirmed to be the *RhtD1b* locus, and *QPh.caas-6A* was found to be the *Rht24* locus (Li et al., 2015a; Tian et al., 2017). Using ‘Nongda3338/Jingdong6’ doubled haploid (DH) population, *QPh.cau-4B.2*, *QPh.cau-4D.1* and *QPh.cau-2D.3* were found to be associated with the dwarfing genes *Rht1*, *Rht2*, and *Rht8*, respectively (Guan et al., 2018).

To date, only several genes for PH have been cloned, which the functions were verified using transgenic technologies. The green revolution genes *Rht-B1* and *Rht-D1* were the first cloned genes. The dwarfing alleles reduced stem growth by stabilizing the encoded DELLA proteins against degradation through interactions with the growth hormone gibberellin (GA) and its receptor (Peng et al., 1999). *Rht-B1* and *Rht-D1* have a variety of alleles, including *Rht-B1c* (*Rht3*), *Rht-D1c* (*Rht10*), *Rht-B1e* (*Rht11*), and *Rht-B1p* (*Rht17*) (Cao et al., 2009; Pearce et al., 2011; Li et al., 2012a, b, 2013; Bazhenov et al., 2015). The *Rht8* gene on chromosome 2DS is considered more influential than the green revolution genes (Botwright et al., 2005; Tian et al., 2017). It was cloned as a *ribonuclease h-like domain* gene using map-based cloning. It controls the PH and spike length via GA biosynthesis (Chai et al., 2022; Xiong et al., 2022). The *TaGA2ox-A14* gene was reported to be a candidate gene of *Rht12* based on fine mapping, expression profiling and GA content analysis (Sun et al., 2019). *GA2oxA13* (previously named *GA2oxA14*) was identified as the causal gene of *Rht12* through the MutChromSeq approach (Buss et al., 2020). Fine mapping revealed that *Rht13* was located on the long arm of chromosome 7B (Ellis et al., 2005). *Rht-B13* encodes *a nucleotide-binding site/leucine-rich repeat* (*NB-LRR*) gene. Autoactivation of *Rht13* leads to the upregulation of pathogenesis-related (PR) genes including class III peroxidases, which may catalyze the crosslinking of cell wall compounds to limit cell elongation and hence reduce height (Borrill et al., 2022). *Rht18* gene was identified by mutagenizing the semidwarf durum cultivar Icaro and generating mutants with a range of tall phenotypes. The increased expression of *GA2oxA9* in *Rht18* results in a reduction of both bioactive GA content and plant height (Ford et al., 2018). *Rht24* was isolated by map-based cloning and found to encode the GA2-oxidase *TaGA2ox-A9* (Tian et al., 2017). *Rht24b* conferred higher expression of *TaGA2ox-A9* in stems, leading to a reduction of bioactive GA in stems but an elevation in leaves at the jointing stage (Tian et al., 2022). Our group identified a candidate gene *ATP-dependent DNA helicase* (*TaDHL-7B*) for PH via QTL mapping and genome-wide association study (GWAS) methods. The knock out mutants of *TaDHL-7B* significantly reduced the PH without a yield penalty (Guo et al., 2022).

In this study, we mapped a QTL *QPh-1B* that included an EST-SSR marker *swes1079*. The full length of cDNA and gDNA were cloned according to the sequence of *swes1079*, which is the orthologous gene of *nonselective hyperosmolality-gate calcium-permeable channel 1.4* (*OSCA1.4*). Then, we demonstrated that *TaOSCA1.4* was associated with PH using a natural population. Finally, we validated that the *TaOSCA1.4* gene controls PH via RNAi technology.

## 2 Materials and methods

### 2.1 Plant materials and trial design

QTL analysis was performed using a set of RILs derived from a cross of ‘Chuan35050 × Shannong 483’ (C35050 × SN483, C/SN-RILs, 131 lines) that constructed began in 1993 (Li et al., 2007). C35050 was a variety from the Southwestern Winter Wheat Region in China. SN483 was a variety of the Huang-huai Winter Wheat Region, China. SN483 was derived from ‘Ai-Meng-Niu’, one of the most well-known germplasms and backbone parents in Chinese wheat breeding programs. ‘Ai-Meng-Niu’ was developed by Shandong Agricultural University in 1980. More than 16 well-known cultivated varieties, which planted on more than 30 million hectares, have been developed from ‘Ai-Meng-Niu’. The C/SN-RILs and their parents were planted at four environments, Tai’an in 1999 (E1), 2000 (E2), 2001 (E3), and Yantai in 2001 (E4), with two replications (Li et al., 2007). The PH for each line and parent were determined by the sample of 10 plants in each replication.

A natural population of 134 current wheat varieties from the Huang-huai Winter Wheat Region of China was used to test the association between the marker and the PH. The field trials were located at the Experimental Station of Shandong Agricultural University (Tai’an, China), the Zibo Academy of Agricultural Sciences (Zibo, China), and the Xinjiang Academy of Agricultural Sciences (Urumqi, China) in the 2012–2013 and 2013–2014 growing seasons under irrigation and rainfed conditions. These environments were noted as Tai’an irrigation (TAI), Zibo irrigation (ZBI), Urumqi irrigation (URI), Tai’an rainfed (TAR), Zibo rainfed (ZBR) and Urumqi rainfed (URR) in 2013 (13) and 2014 (14), respectively. Seeds were sown on October 5–10, and plants were harvested on June 10–15 of the next year. Each plot consisted of three rows, which were 1.5 m long and spaced 25 cm apart, with two repetitions. Fifty seeds were planted in each row.

### 2.2 QTL analysis of the C/SN-RILs

A genetic map (Wang et al., 2011) was used to carry out the QTL analysis. The map consisted of 719 markers on 21 chromosomes, with a total length of 4,008.4 cM. The Windows QTL Cartographer 2.5 software (http://statgen.ncsu.edu/qtlcart/WQTLCart.htm) was used to perform the QTL mapping. The parameters were as follows: composite interval mapping (CIM), “model 6 standard analysis”, a walk speed of 1 cM, “forward and backward” regression, up to five control markers, and a blocked window size of 10 cM. The threshold for declaring the presence of a significant QTL was defined by 1,000 permutations at p ≤ 0.05 (Churchill and Doerge, 1994). The LOD threshold for declaring a significant QTL was an LOD≥3.0 (He et al., 2020; Li et al., 2021; Xu et al., 2021).

### 2.3 DNA/RNA extraction

For the parents of the C/SN-RIL population and the variety ‘Fielder’ (wild-type, WT), total DNA was extracted using a DNA extraction kit (Tiangen, Beijing, China). The quality and concentration of DNA were determined using a NanoDrop 2000c spectrophotometer (Thermo, Wilmington, DE, USA). Total RNA was extracted using the RNAprep Pure Plant Kit (TIANGEN, Beijing, China). The RNA purity was checked using a nanophotometer spectrophotometer (IMPLEN, CA, USA). The RNA integrity was assessed using an RNA Nano 6000 Assay Kit for the Agilent Bioanalyzer 2100 system (Agilent Technologies, CA, USA). Reverse transcription into cDNA was performed according to the manufacturer’s protocol.

### 2.4 Amplification and chromosomal localization of candidate genes

The sequence of expressed sequence tag - simple sequence repeat (EST-SSR) marker *swes1079* (GenBank ID: CJ623047.1) was used as a query sequence to screen the wheat EST database on GenBank (http://www.ncbi.nlm.nih.gov). We selected the highly overlapping EST sequence with those about *swes1079* and used DNAMAN software (http://www.lynnon.com/) to get the splicing sequence. Using the splicing sequence as the starting sequence to continue search the wheat EST datebase and further extension. Then, a putative gene cDNA sequence was assembled. After that, we identified the Open Read Frame (ORF) using the ORF Finder program (http://www.ncbi.nlm.nih.gov/gorf/gorf.html). Primer pairs was designed using the Primer Premier 5.0 software to produce the cDNA and gDNA sequence based the ORF. The primers were as follows:

TaOSCA1.4-F: ATGGCGACGCTGCAGGA
TaOSCA1.4-R: TCAATGATCGACTCCAGGGT

Genome-specific primers were designed according to the difference sites of the three gDNA sequences of *TaOSCA1.4*, and the chromosomal localization was carried out using Chinese Spring nullisomic-tetrasomic lines. The primers were as follows:

TaOSCA1.4–1AF: CGCGTCTACTTCCCCAA
TaOSCA1.4–1AR: ATCTTTTCTCAGGTTCAGTACG
TaOSCA1.4–1BF: GAAGATGGGGTGCAGGTGA
TaOSCA1.4–1BR: GAATACCAAGGACCATTGGAG
TaOSCA1.4–1DF: TTCCGGTCAATGTCTCTGAT
TaOSCA1.4–1DR: ATCAAGTTCGCTAATTCTAGCC

### 2.5 Knockdown of the *TaOSCA1.4* gene by RNAi

The following 94 bp nucleotide sequence, located in the seventh exon, was used as the interference fragment:

TTATGCCAGTTTCTTTTGTGACATTTGACTCAAGATGGGGTGCTGCTGTATGTGCACAGACACAACAGTCAAAGAATCCCACACAATGGCTGAC

The DNA was recovered using an agar-gel DNA recovery kit (Tiangen, Beijing, China). The BP reaction system consisted of 150 ng attB-PCR product, 150 ng pDONR Zeo vector, and 2 μl BP Clonase II enzyme mix in a 10 μl system was supplemented with RNase-free water. The mixture was incubated at 25°C for 2 h. The reaction was terminated by adding 1 μL of proteinase K and incubating at 37°C for 10 min. The reaction mixture was subsequently transformed into *E. coli*. Pick the clone and verify the sequence. The LR reaction mixture consisted of 150 ng of the entry-target fragment, 150 ng of the pANDA vector, 2 μl of the LR Clonase II enzyme mixture, and 10 μl of system supplemented with RNase-free water. The mixture was incubated at 25°C for 2 h. The reaction was terminated by adding 1 μL of proteinase K and incubating at 37°C for 10 min. The reaction mixture was subsequently transformed into *E. coli*. Pick the clone and verify the sequence. The plasmid was subsequently transformed into an *Agrobacterium* strain, after which the wheat genetic transformation experiment and wheat transgenic platform were established by the Crop Research Institute, Shandong Academy of Agricultural Sciences, China. The genome of transgenic wheat was extracted, gene expansion was determined by primers, and the bar gene and target gene were detected via agarose gel electrophoresis. Positive transgenic plants were detected via PCR and 1% agarose gel electrophoresis.

### 2.6 qRT−PCR, sequence characteristics, and bioinformatics analysis

The transcript levels of *TaOSCA1.4* in Fielder organs were analyzed via qRT−PCR. The primers used were as follows:

TaOSCA1.4–1A-qRTF: CAAAGAGTACAGTAATGTGGCC,
TaOSCA1.4–1A-qRTR: AGATGTCGAATGGCTTGAC,
TaOSCA1.4–1B-qRTF: GAGATTACATTTCCTGGCTTCC,
TaOSCA1.4–1B-qRTF: CGGAAGAACTCATCAACTGC,
TaOSCA1.4–1D-qRTF: ACCACTATCTTGGTCAGCAGT
TaOSCA1.4–1D-qRTF: CAGGATGCCTTTCAAACTTC.

The first-strand cDNAs were synthesized from 2 µg of RNA per sample using TransScript One-Step gDNA Removal and cDNA Synthesis SuperMix (TransGen, Beijing, China). The qRT−PCR aplifications were performed as described by Zhao et al. (2020). Amplification of TaActin was used as an internal control for data normalization. The experiments were independently replicated three times under identical conditions. The complete alignment of multiple coding sequences and translations of nucleotides into amino acid sequences were performed using the DNAMAN program (version 5.2.2; Lynnon Biosoft, Canada). The prediction of the transmembrane structure was performed using DeepTMHMM (https://dtu.biolib.com/DeepTMHMM). Collinearity analysis was performed using the Triticeae-Gene Tribe (TGT, http://wheat.cau.edu.cn/TGT/).

### 2.7 Cytological analysis

For the Fielder and RNAi plants, the top of the first internode from the top of the main stem was taken, and 3 biological replicates were performed. Semithin sections were prepared by fixation, dehydration, paraffin impregnation and embedding, dewaxing, plant saffron stain, and then decolorization with an alcohol gradient (50%, 70% and 80%). After the plants were dyed with plant solid green dye solution and dehydrated with anhydrous ethanol, the samples were cleared in xylene and sealed with neutral gum. A Nikon Eclipse Ci-L photographic microscope was used for 400x imaging. After the imaging was completed, Image-Pro Plus 6.0 analysis software was used to count the number of cells, measure the area of cell tissue in the visual field, and calculate the number of cells per unit area = the number of cells/the area of cell tissue in the visual field.

### 2.8 Subcellular localization

According to the sequence structure characteristics of pBI121-GFP vector, a seamless cloning joint was designed in front of upstream and downstream primers, and a seamless cloning primer was designed:

YXB-3-F: gagagaacacgggggactctagaATGGCGACGCTGCAGG
YXB-3-R: cataagggacrgaccacccggGTGCTCGACTCCAT.

Synthetized Fielder cDNA was used as a template to amplify the cDNA sequences. The upstream primers anneal beginning at the start codon (ATG), and the downstream primers anneal adjacent to the stop codon (not including the stop codon). The reaction system consisted of 5 μl of buffer, 2 μl of cDNA, 0.4 μl of YXB-3-F, 0.4 μl of YXB-3-R, 1 μl of ApexHF HS DNA Polymerase CL, and 1.2 μl of RNase-free water. The PCR procedure was as follows: predenaturation at 94°C for 2 min, followed by 33 cycles of denaturation at 94°C for 30 s, annealing at 60°C for 30 s, and extension at 72°C for 3 min, with a final extension at 72°C for 5 min.

The pBI121-GFP carrier plasmid was double digested with *XmaI* and *XbaI*. The enzyme digestion reactivity was as follows: 5 μl CutSmart Buffer, 33 μl ddH_2_O, 1 μl *XmaI*, 1 μl *XbaI*, and 10 μl pBI121-GFP. After overnight digestion at 37°C, carrier attachment was performed according to the instructions of the LightNingTM DNA Assembly Mix Plus Kit. The GFP fusion vector was subsequently transformed into Trans1-T1 competent *E. coli* cells, and positive cloning detection primers were designed based on the pBI121-GFP vector sequence:

GFP-F: GTTCCAACCACGTCTTCAAAGC;
GFP-R: CTCGCCGGACACGCTGAACT.

The positive clone bacteria were screened and sent to Sangon Biotech (Shanghai) Co., Ltd for sequencing to verify the correctness of the sequence.

The GFP fusion vector and blank plasmid pBI121-GFP were subsequently transformed into Super EHA105 receptive cells. The specific procedures used referenced the manufacturer’s instructions for the Super EHA105 receptive cells. GFP-F/R was used to detect positive clones, and the strains were preserved.

## 3 Results

### 3.1 Acquisition of a candidate gene *TaOSCA1.4* for PH from a QTL

#### 3.1.1 QTL location of *QPh-1B*

**Figure 1:**
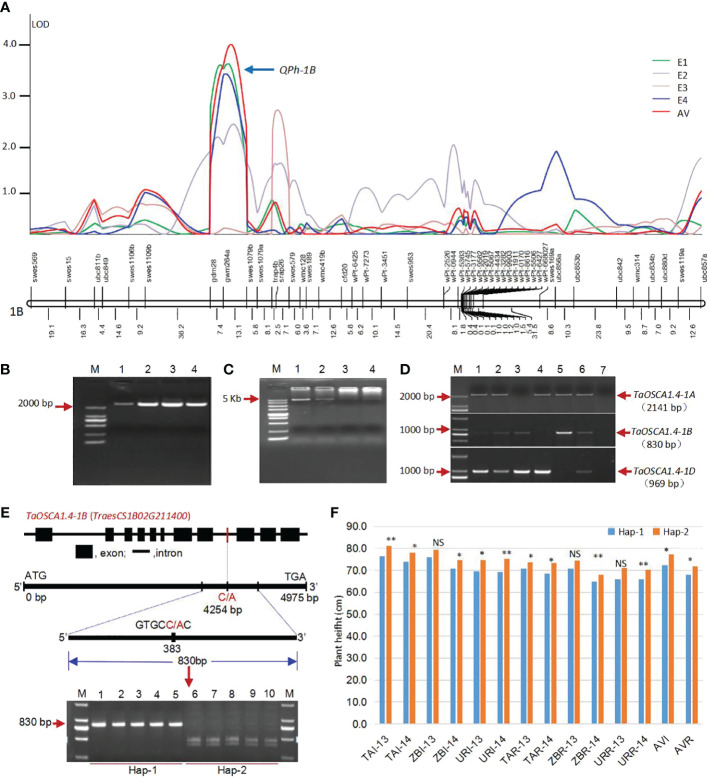
Acquisition of the candidate gene *TaOSCA1.4* for PH. **(A)** QTL location for PH on chromosome 1B under four environments using a genetic map of DNA markers (Wang et al., 2011). **(B)** Full-length of cDNA sequences for *TaOSCA1.4*. M, Marker; 1, Wheat variety Tainong18; 2, Wheat variety Linmai6; 3, Wheat variety Chuan35050; 4, Wheat variety Shannong483. **(C)** Full-length of DNA sequences for *TaOSCA1.4*. M, Marker; 1, Wheat variety Tainong18; 2, Wheat variety Linmai6; 3, Wheat variety Chuan35050; 4, Wheat variety Shannong483. **(D)** Chromosome location of *TaOSCA1.4* homologous genes using Chinese Spring nullisomic-tetrasomic lines. M, Marker; 1, Wheat variety Tainong18; 2, Wheat variety Linmai6; 3, N1AT1D (Nullisomic 1A-tetrasomic 1D); 4, N1BT1D; 5, N1DT1B; 6, Chinese Spring; 7, ddH_2_O. **(E)** Structure of the candidate gene *TaOSCA1.4–1B* (*TraesCS1B02G211400*) and the development of a cleaved amplified polymorphism sequence (CAPS) marker. **(F)** Differences of the PH in the natural population which scanned using the CAPS marker. *, P ≤ 0.05; **, P ≤ 0.01; NS, not significant.

QTL analysis was conducted using C/SN-RIL populations, and a stable QTL for PH, *QPh-1B*, was located on chromosome 1B under two environments (E1 and E4) and average value (AV). This QTL included three molecular markers *gdm28*, *gwm264* and *swes1079* (**Figure 1A**). The markers *gdm28* and *gwm264* was located in noncoding regions. The *swes1079* is a functional EST-SSR marker developed by our research group (Chen et al., 2005).

#### 3.1.2 cDNA and gDNA cloning and chromosomal location of *TaOSCA1.4*

According to the results of searching NCBI database, six wheat ESTs (GenBank ID: *CJ623047.1*, *BJ248974.1*, *CJ730580*, *BQ238836.1*, *CJ726913.1*, *GH731252.1*) were obtained and assembled into a putative cDNA sequence. The cDNA and corresponding gDNA sequences were amplified (**Figures 1B, C**). It is the previously cloned orthologous gene of *nonselective hyperosmolality-gate calcium-permeable channel 1.4* (*OSCA1.4*) in rice, which was specifically downregulated by drought stress and upregulated by ABA treatment by expression profiles (Li et al., 2015b) (**Supplementary Figure 1**). Therefore, we named this gene as *TaOSCA1.4*. Based on the three genome-specific primer pairs, the *TaOSCA1.4* genes were located on chromosomes 1A, 1B and 1D using the Chinese Spring nullisomic-tetrasomic lines (**Figure 1D**). For *TaOSCA1.4–1A*, the full length of gDNA is 5142 bp and cDNA is 2418 bp, encoding 805 amino acids (AAs). For *TaOSCA1.4–1B*, the full length of gDNA is 4975 bp and cDNA is 2406 bp, encoding 801 AAs. For *TaOSCA1.4–1D*, the full length of gDNA is 4961 bp and cDNA is 2409 bp, encoding 802 AAs. After the RefSeq v1.1 genome released in 2018 (IWGSC, 2018), we found that the *TaOSCA1.4–1B* gene is *TraesCS1B02G211400*. Its homologous genes on chromosomes 1A and 1D were *TraesCS1A02G196800* and *TraesCS1D02G200300*, respectively. The DNA sequence similarities of *TaOSCA1.4* in between A and B, A and D, and B and D were 84.29%, 77.18%, 79.56%, respectively. The AA sequence similarities between A and B, A and D, and B and D were 97.76%, 97.88%, 98.63%, respectively.

#### 3.1.3 Associated analysis between *TaOSCA1.4* and PH using a natural population

To further show the relationship between *TaOSCA1.4* and PH, we developed a cleaved amplified polymorphism sequence (CAPS) marker from *TaOSCA1.4–1B* to analyze the variation across a natural population. The CAPS marker divided the natural population into two haplotypes, Hap-1 (830 bp) and Hap-2 (447 and 383 bp) (**Figure 1E**). PH was significantly different between the two haplotypes under most of the 12 environments and the average values of irrigation (AVI) and rainfed (AVR) conditions (**Figure 1F**). This result further demonstrated that *TaOSCA1.4* was associated with PH.

### 3.2 Functional confirmation of the *TaOSCA1.4* gene using RNAi technology

**Figure 2:**
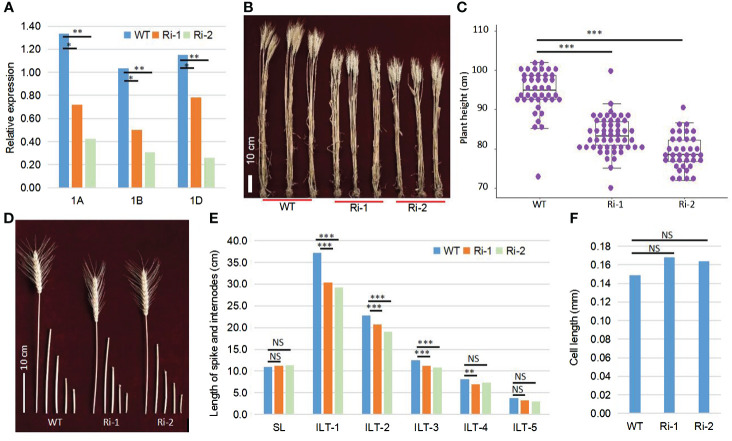
Function confirmation of the *TaOSCA1.4* gene using the RNAi system. **(A)**, Expression levels of three homologous genes *TaOSCA1.4–1A* (1A)*, TaOSCA1.4–1B* (1B) and *TaOSCA1.4–1D* (1D) in WT (Fielder), Ri-1 and Ri-2 by qRT-PCR. **(B)** Picture of the plant for WT, Ri-1 and Ri-2. **(C)** Boxplot of the plant height for WT, Ri-1 and Ri-2. **(D)** Picture of spikes and internodes for WT, Ri-1 and Ri-2. **(E)** Difference for the length of spikes and internodes between WT and Ri-1 and Ri-2. **(F)**, Cell length of the first internode from top for WT and Ri-1 and Ri-2. *, *P ≤* 0.05; **, *P ≤* 0.01; ***, *P ≤* 0.001; NS, not significant.

To obtain the conclusive evidence of the relationship between *TaOSCA1.4* and PH, we performed RNA interference analysis of the *TaOSCA1.4–1A*, *TaOSCA1.4–1B* and *TaOSCA1.4–1D* genes using the RNAi system. We planted the WT and RNAi mutant plants of the T_4_ generation in the field. The qRT−PCR results showed that the expression levels of three homologous genes of *TaOSCA1.4* in RNAi lines 1 (Ri-1) and 2 (Ri-2) were significantly decreased (**Figure 2A**; **Supplementary Table 1**). According to the results of field trials, the average PHs of the WT, Ri-1 and Ri-2 were 94.6, 83.6 and 79.2 cm, respectively, with significant differences between the WT and Ri-1 and Ri-2 (**Figures 2B, C**). Compared with those of the WT plants, the heights of Ri-1 and Ri-2 were reduced by 11.0 and 15.4 cm, respectively (**Supplementary Table 2**). These results indicated that the *TaOSCA1.4* gene controls PH. The spike length (SL) was not significantly different between the WT and Ri-1 and Ri-2. The first, second, and third internode length from the top (ILT-1. ILT-2 and ILT-3) decreased by 6.8, 2.1, and 1.3 cm, respectively, in Ri-1; and decreased by 8.0, 3.8, and 1.8 cm, respectively, in Ri-2. These three internode lengths were significantly different from those in the WT (**Figures 2D, E**; **Supplementary Table 2**).

We observed longitudinal sections of WT and the RNAi lines using the uppermost internodes at the vegetative stage. The cell lengths of the WT, Ri-1 and Ri-2 were 0.149, 0.168 and 0.164 mm, respectively. The cell lengths between WT and Ri-1 and Ri-2 were not significantly different (**Figure 2F**; **Supplementary Table 3**).

### 3.3 Structure, expression, evolution and subcellular localization of *TaOSCA1.4*

For DNA sequences of *TaOSCA1.4–1B* between the parents (C35050 and SN483) of the RILs, there are two SNPs (at positions 78 and 4870 bp) and one InDel (6 bp deletion at position 4768 in SN483) in the exons; and are six additional SNPs (at positions 618, 783, 1029, 2817, 3859 and 4255 bp) and four InDels (deletion of 3 bp at position 1110, 8 bp at position 2459 and 1 bp at position 3297 SN 483; and deletion of 1 bp at position 2866 in C35050) in the introns (**Supplementary Figure 2**). For the AA sequences, two adjacent Q residues were deleted at positions 739 and 740 in SN 483, and an R substitution occurred in C35050 at position 759 (this residue is Q in SN 483) (**Supplementary Figure 3**). For *TaOSCA1.4–1A* and *TaOSCA1.4–1D*, the DNA sequences are not differences between the two parents.

**Figure 3:**
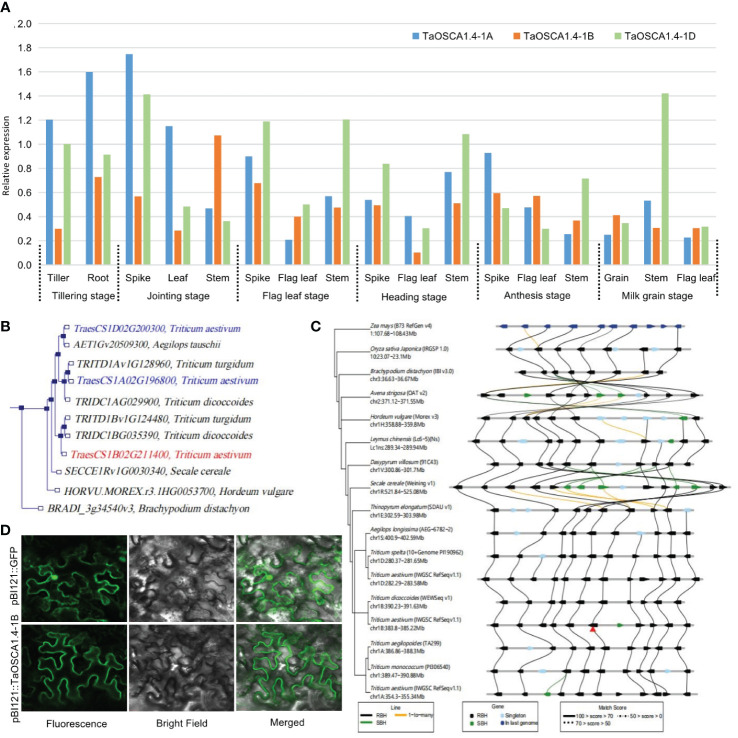
Characteristics of the *TaOSCA1.4.* **(A)** Expression levels in different tissues and periods for the three homologous genes *TaOSCA1.4–1A, TaOSCA1.4–1B* and *TaOSCA1.4–1D* in WT by qRT-PCR. **(B)** Phylogenetic tree for three homologous genes *TaOSCA1.4–1A, TaOSCA1.4–1B* and *TaOSCA1.4–1D*. **(C)** Collinearity analysis of *TaOSCA1.4*. **(D)** Result of subcellular localization for *TaOSCA1.4–1B*.

The expression levels of *TaOSCA1.4* in different tissues and at different periods determined by qRT-PCR showed that *TaOSCA1.4* is a constitutively expressed gene. For *TaOSCA1.4–1A*, the highest expression is in the spike at the joining stage. For *TaOSCA1.4–1B*, the highest expression is in the stem at the joining stage. For *TaOSCA1.4–1D*, the highest expression is in the stem at milk grain stage (**Figure 3A**).

A phylogenetic tree generated using *Ensemble* (http://plants.ensembl.org/Triticum_aestivum/) revealed that *TaOSCA1.4–1B* (*TraesCS1B02G211400*), *TaOSCA1.4–1A* (*TraesCS1A02G196800*) and *TaOSCA1.4–1D* (*TraesCS1D02G200300*) were on different branches (**Figure 3B**). *TaOSCA1.4–1B* is closely related to *TRITD1Bv1G124480* of *Triticum turgidum* and *TRIDC1BG035390* of *Triticum dicoccoides*. *TaOSCA1.4–1A* is closely related to *TRITD1Av1G128960* of *Triticum turgidum* and *TRIDC1AG029900* of *Triticum dicoccoides. TaOSCA1.4–1D* is closely related to *AET1Gv20509300* of *Aegilops tauschii*. A collinearity analysis using *Triticeae-GeneTribe* (http://wheat.cau.edu.cn/TGT/) revealed that the *TaOSCA1.4–1B* region was conserved between genomes A, B, D, and E, but an inversion occurred in *Secale cereale* (genome R) (**Figure 3C**).

Subcellular localization was predicted with a transmembrane domain hidden Markov model, which suggested that the *TaOSCA1.4–1B* protein is a typical transmembrane protein with eight transmembrane domains. According to the subcellular localization results, the green fluorescence presented by the protein encoded by the target gene *TaOSCA1.4–1B* had a strong signal only on the cell membrane, and no signal was detected in the rest of the cell. This result suggested that the *TaOSCA1.4* protein localizes to the cell membrane (**Figure 3D**).

## 4 Discussion

Wheat is an allohexaploid species with a large and highly complex genome that severely restricts the isolation of genes via the classical method of map-based cloning. Only a few genes for PH, including *Rht8* (Chai et al., 2022; Xiong et al., 2022), *Rht13* (Borrill et al., 2022)*, Rht23* (Zhao et al., 2018) and *Rht24* (Tian et al., 2022), have been cloned via map-based cloning. On the other hand, a great number of QTLs for PH have been identified (Saini et al., 2022; Singh et al., 2022). However, the intervals of these QTLs detected using a high-density genetic map of DNA markers are usually include dozens of genes or even more (Choulet et al., 2014). Due to these DNA markers being mainly located in noncoding regions, determining how to use QTLs to clone genes is a challenge. We previously developed EST-SSR markers (Chen et al., 2005), which represent a partial sequence of a gene. We mapped these markers to construct a genetic map (Wang et al., 2011). In this study, we found that the QTL *QPh-1B* included the EST-SSR marker *swes1079*, which provided the possibility for identifying the related gene *TaOSCA1.4*. We then proved this possibility by identifying candidate genes in populations with natural variation. Finally, we validated that *TaOSCA1.4* regulate PH via RNAi technology. By the above process, we successfully produced a cloned gene from a QTL. This provides a method for gene cloning. Based on this idea, we constructed a map of unigenes using an RIL population and cloned seven yield component trait genes, *TaIFABPL, TaDdRp, TaRLK, TaTD, TaTFC3, TaKMT* and *TaSPL17* (Zhang et al., 2024), a plant height gene *TaDHL* (Guo et al., 2022), and a quality gene *XIP* (Sun et al., 2022) from QTLs. This approach could be an effective method for isolating genes originally from wheat.

The studies of *OSCA* gene family were focused the expression profiling under osmotic stress in *Arabidopsis*, rice, wheat and barley, etc. (Li et al., 2015b; Zhai et al., 2020; Tong et al., 2021; Kaur et al., 2022; She et al., 2022). In wheat, Tong et al. (2021) identified 42 *OSCA* members. The expression profiles showed that 15 *TaOSCA* members responded to PEG treatment, while *TaOSCA12*/-*39* responded simultaneously to PEG and ABA. Kaur et al. (2022) also identified 42 *OSCA* genes. They found that a total of 31 *TaOSCA* genes displayed differential expression in response to the drought, heat, and combined heat–drought stress conditions, and 29 *TaOSCA* genes showed differential expression in response to salt stress. No report was found that *OSCA* genes regulating the plant height. Using RNAi technology, we obtained the conclusive evidence that *TaOSCA1.4* regulate PH. So, we regard that the *TaOSCA1.4* is novel reduced-height (*Rht*) gene in wheat.

*OSCA* genes regulate intracellular Ca^2+^ concentration and play a key role in sensing exogenous and endogenous osmotic changes and regulating plant growth and development. In *Arabidopsis*, the *osca1* mutant exhibited a decreased calcium (Ca^2+^) concentration under sorbitol or mannitol treatment, inhibited stomatal closure and root growth, indicating that *OSCA1* is a plant osmoreceptor (Yuan et al., 2014). In rice, *OsOSCA1.1* mediates OICIcyt and SICIcyt in roots, which are critical for stomatal closure, plant survival, and gene expression in shoots, in response to hyperosmolality and the salt stress treatment of roots (Han et al., 2022). When plants are subjected to external stress, Ca^2+^ flows from high concentrations of extracellular and intracellular calcium reservoirs into the cytoplasm, resulting in an instantaneous increase in the concentration of Ca^2+^ in the cytoplasm and the generation of calcium signals. The calcium signal is then recognized by three major Ca^2+^ receptor proteins: calmodulin (CaM) (Dong and Zhang, 2001), calcineurin B-like protein (CBLS) (Desikan et al., 2006; Shabala et al., 2018), and calcium-dependent protein kinases (CDPKs) (Cheng et al., 2002; Chen et al., 2013) and triggers a series of physiological activities. We hypothesized that knock down mutants of *TaOSCA1.4* gene reduced regulatory ability of Ca^2+^, thus reducing the plant height.

Among the cloned *Rht* genes, *Rht-B1* and *Rht-D1* (Peng et al., 1999), *Rht8* (Chai et al., 2022; Xiong et al., 2022), *Rht12* (Buss et al., 2020), *Rht18* (Ford et al., 2018), *Rht24* (Tian et al., 2022) are involved in GA signaling or metabolism pathway. *Rht13*, an NB-LRR gene, is not directly involved in GA pathway, and the same mutation in the tomato protein I-2, which impeded ATP hydrolysis and promoted an ATP-bound active form of the protein (Borrill et al., 2022). ZnF-B acts as a brassinosteroid (BR) signaling activator to facilitate proteasomal destruction of the BR signaling repressor TaBKI1, and loss of ZnF stabilizes TaBKI1 to block BR signaling transduction. The deletion of *ZnF-B* induced the semi-dwarf trait in the absence of the *Rht-B1b* and *Rht-D1b* alleles through BR perception (Song et al., 2023b). We previous cloned an *Rht* gene *TaDHL* is not directly hormone pathway (Guo et al., 2022). In this study, *TaOSCA1.4* gene regulates intracellular Ca^2+^ concentration. Furthermore, the cell lengths of the knock down mutants is not significantly different than that of WT. We speculate that *TaOSCA1.4* gene is not directly associated with GA, which should be a novel mechanism for a wheat *Rht* gene.

## Data availability statement

The datasets presented in this study can be found in online repositories. The names of the repository/repositories and accession number(s) can be found in the article/**Supplementary Material**.

## Author contributions

SL: Writing – original draft, Writing – review & editing, Supervision. GL: Writing – original draft, Data curation, Investigation, Methodology. XJ: Writing – original draft, Data curation, Investigation, Methodology. HW: Investigation, Methodology, Writing – original draft. YW: Data curation, Writing – original draft. QW: Data curation, Software, Writing – original draft. HMW: Investigation, Writing – original draft, Data curation. FJ: Investigation, Writing – original draft. YM: Investigation, Writing – original draft. YA: Methodology, Writing – original draft. MZ: Data curation, Software, Writing – original draft. YG: Writing – original draft, Investigation, Methodology.
