# Supplementary material for: Cloning a novel reduced-height (Rht) gene TaOSCA1.4 from a QTL in wheat
Source: Front Plant Sci. 2024 May 16;15:1381243. doi: 10.3389/fpls.2024.1381243 (PMC11137288; doi:10.3389/fpls.2024.1381243)
Supplement: Supplementary file 4 [file DataSheet_1.pdf]

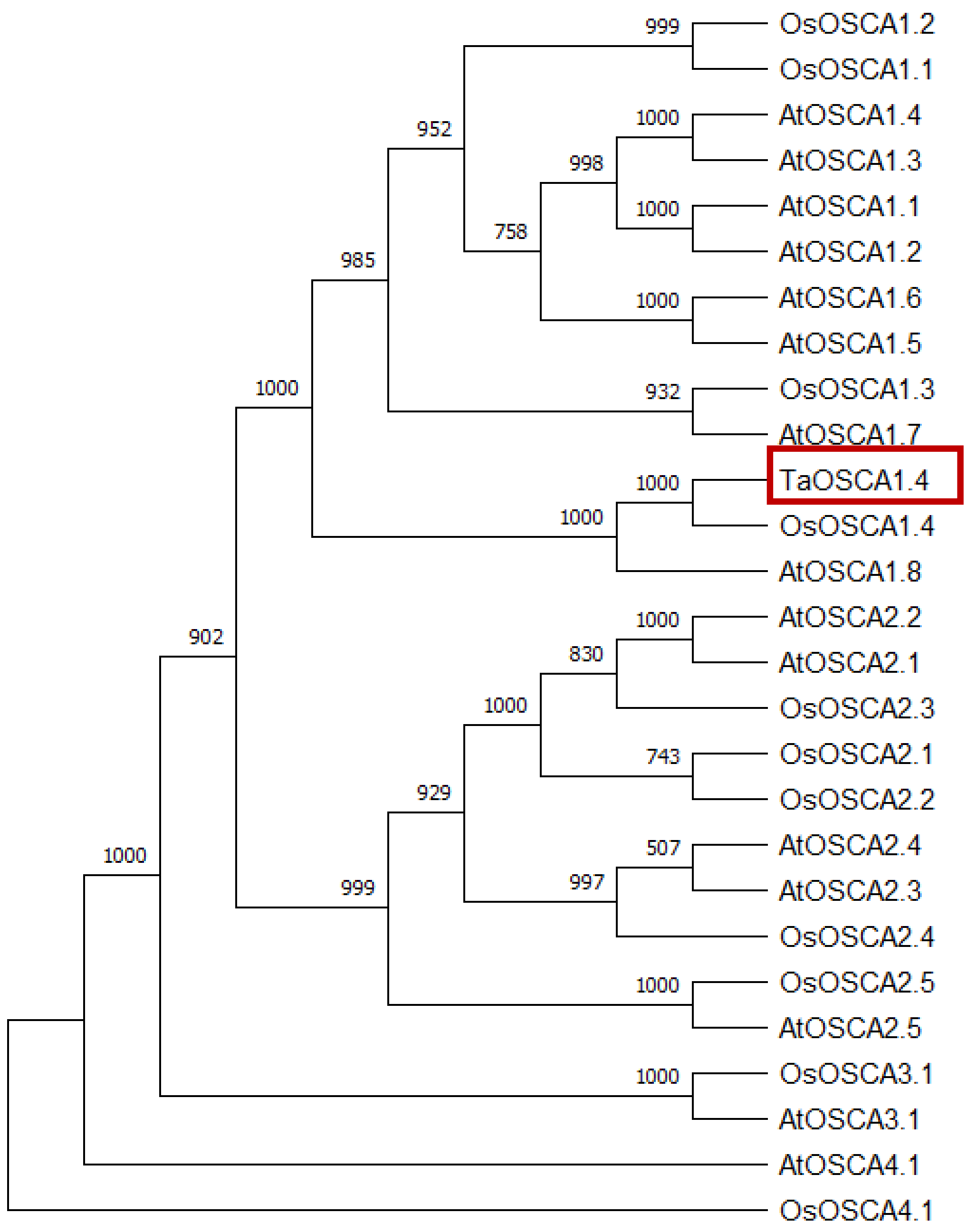

Supplementary Figure 1. The phylogenetic tree of *TaOSCA1.4* and *OSCA* family members from *Arabidopsis* and *Rice*.
