# Supplementary material for: Cloning a novel reduced-height (Rht) gene TaOSCA1.4 from a QTL in wheat
Source: Front Plant Sci. 2024 May 16;15:1381243. doi: 10.3389/fpls.2024.1381243 (PMC11137288; doi:10.3389/fpls.2024.1381243)
Supplement: Supplementary file 5 [file DataSheet_2.pdf]

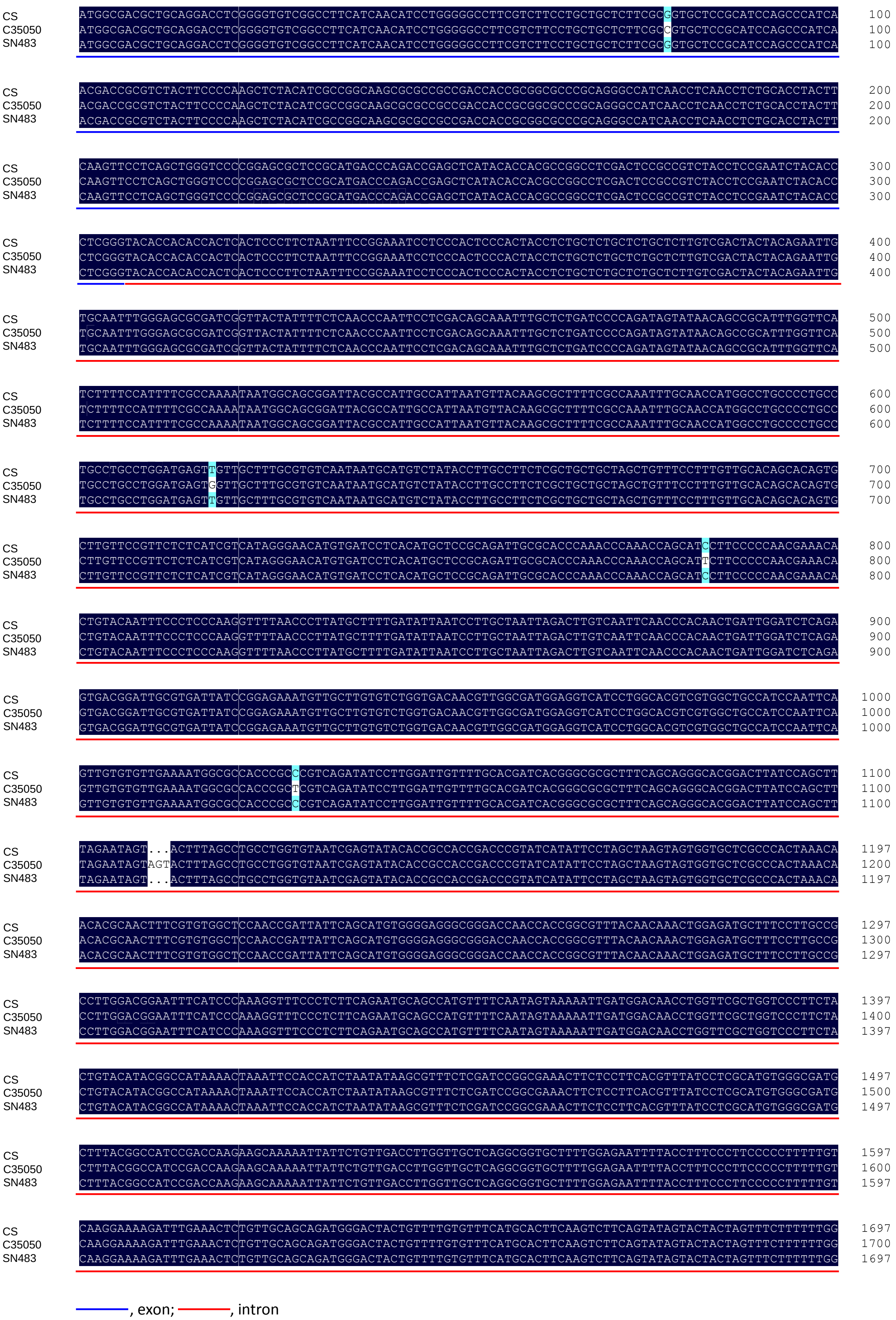

Supplementary Figure 2. Difference of DNA sequences between C35050 and SN483 for *TaOSCA1.4-1B* gene.

```
CS       TGGCTATTTTACAGGTTCAATTTGAGCCCTCTTTTTCATACACTTGACTATTTTTTTTCCTCTGGTCTCACTTGCAGCCTCAAGATTTTTTTGCCCGTCA 1797
C35050   TGGCTATTTTACAGGTTCAATTTGAGCCCTCTTTTTCATACACTTGACTATTTTTTTTCCTCTGGTCTCACTTGCAGCCTCAAGATTTTTTTGCCCGTCA 1800
SN483    TGGCTATTTTACAGGTTCAATTTGAGCCCTCTTTTTCATACACTTGACTATTTTTTTTCCTCTGGTCTCACTTGCAGCCTCAAGATTTTTTTGCCCGTCA 1797

CS       TGGTTGTCGCCTTGCTGGTTCTCATTCCGGTCAATGTCTCTGGAGGGACACTACTGAACCTGAGAAAAGATGTTATGTTCAGTGATATTGATAAGCTGTC 1897
C35050   TGGTTGTCGCCTTGCTGGTTCTCATTCCGGTCAATGTCTCTGGAGGGACACTACTGAACCTGAGAAAAGATGTTATGTTCAGTGATATTGATAAGCTGTC 1900
SN483    TGGTTGTCGCCTTGCTGGTTCTCATTCCGGTCAATGTCTCTGGAGGGACACTACTGAACCTGAGAAAAGATGTTATGTTCAGTGATATTGATAAGCTGTC 1897

CS       CATATCAAATGTTAGCCCCGGCTCCAACAGGTAATCTTCAGTAACTTATGAACTCTGAATTTGCCAGTTCATTTTTTATCCTAACACAAAGTCAGAACAT 1997
C35050   CATATCAAATGTTAGCCCCGGCTCCAACAGGTAATCTTCAGTAACTTATGAACTCTGAATTTGCCAGTTCATTTTTTATCCTAACACAAAGTCAGAACAT 2000
SN483    CATATCAAATGTTAGCCCCGGCTCCAACAGGTAATCTTCAGTAACTTATGAACTCTGAATTTGCCAGTTCATTTTTTATCCTAACACAAAGTCAGAACAT 1997

CS       TGTTCTTTAAAACTCTACATTGTTCTAGGTTGTATGTTCTGATTTGTAATAGTACTCTAAAATGTTGACCCTTGACATCCCACTTCATTTCAGGTTCTTT 2097
C35050   TGTTCTTTAAAACTCTACATTGTTCTAGGTTGTATGTTCTGATTTGTAATAGTACTCTAAAATGTTGACCCTTGACATCCCACTTCATTTCAGGTTCTTT 2100
SN483    TGTTCTTTAAAACTCTACATTGTTCTAGGTTGTATGTTCTGATTTGTAATAGTACTCTAAAATGTTGACCCTTGACATCCCACTTCATTTCAGGTTCTTT 2097

CS       ATTCATCTTCTAATGGCGTATGTGTTCACATTTTGGACGTGCTTTATGCTGTACAAAGAGTACAGTAATGTGGCATTCATGAGATTACATTTCCTGGCTT 2197
C35050   ATTCATCTTCTAATGGCGTATGTGTTCACATTTTGGACGTGCTTTATGCTGTACAAAGAGTACAGTAATGTGGCATTCATGAGATTACATTTCCTGGCTT 2200
SN483    ATTCATCTTCTAATGGCGTATGTGTTCACATTTTGGACGTGCTTTATGCTGTACAAAGAGTACAGTAATGTGGCATTCATGAGATTACATTTCCTGGCTT 2197

CS       CCCAGAAACGTTGTGCTGATCAGTTCACTGTAAGTACATTAACAAAGTTTGCTACTCGTCGAGAGCTGTCCTACTGTAGCATTTAATAATAATAATAAAA 2297
C35050   CCCAGAAACGTTGTGCTGATCAGTTCACTGTAAGTACATTAACAAAGTTTGCTACTCGTCGAGAGCTGTCCTACTGTAGCATTTAATAATAATAATAAAA 2300
SN483    CCCAGAAACGTTGTGCTGATCAGTTCACTGTAAGTACATTAACAAAGTTTGCTACTCGTCGAGAGCTGTCCTACTGTAGCATTTAATAATAATAATAAAA 2297

CS       ACAATTCCAGGTGATTGTTAGAAACATACCTCGTGTTTCAAGCCATTCGACGTCTGAGGCAGTTGATGAGTTCTTCCGCAGGAACCACCCAGATCACTAT 2397
C35050   ACAATTCCAGGTGATTGTTAGAAACATACCTCGTGTTTCAAGCCATTCGACGTCTGAGGCAGTTGATGAGTTCTTCCGCAGGAACCACCCAGATCACTAT 2400
SN483    ACAATTCCAGGTGATTGTTAGAAACATACCTCGTGTTTCAAGCCATTCGACGTCTGAGGCAGTTGATGAGTTCTTCCGCAGGAACCACCCAGATCACTAT 2397

CS       CTTGGTCAGCAGGTATACTATCCTCATTCTAGTTGATTTTTACACACACACACACACACAC........ACACTTGCAAATAACGGTTGCCGGCTAGTGT 2489
C35050   CTTGGTCAGCAGGTATACTATCCTCATTCTAGTTGATTTTTACACACACACACACACACACACACACGCACACTTGCAAATAACGGTTGCCGGCTAGTGT 2500
SN483    CTTGGTCAGCAGGTATACTATCCTCATTCTAGTTGATTTTTACACACACACACACACACAC........ACACTTGCAAATAACGGTTGCCGGCTAGTGT 2489

CS       TTTAATTCTAGAGCTAACCTTATTTTTTTATTTCAGGCTGTTTACAATGCAAACCGGTATGCTAAACTTGTAAAGCAAAAGGAGAGACTTCAAAACTGGCT 2589
C35050   TTTAATTCTAGAGCTAACCTTATTTTTTTATTTCAGGCTGTTTACAATGCAAACCGGTATGCTAAACTTGTAAAGCAAAAGGAGAGACTTCAAAACTGGCT 2600
SN483    TTTAATTCTAGAGCTAACCTTATTTTTTTATTTCAGGCTGTTTACAATGCAAACCGGTATGCTAAACTTGTAAAGCAAAAGGAGAGACTTCAAAACTGGCT 2589

CS       GGATTACTACCAGCTAAAGTTTGAAAGGCATCCTGAGAAAAGACCAACTGGAAGGGTAATGTTCCAATCATTGCTTTTGTTATTTTGGACATGTGCATAT 2689
C35050   GGATTACTACCAGCTAAAGTTTGAAAGGCATCCTGAGAAAAGACCAACTGGAAGGGTAATGTTCCAATCATTGCTTTTGTTATTTTGGACATGTGCATAT 2700
SN483    GGATTACTACCAGCTAAAGTTTGAAAGGCATCCTGAGAAAAGACCAACTGGAAGGGTAATGTTCCAATCATTGCTTTTGTTATTTTGGACATGTGCATAT 2689

CS       AAACTGCTAACCTTATTCATGGTATTTCAGACAGGATGCTTTGGTTTCTGTGGTAGACAAGTGGATCAAATTGACTACTACCGGGCTAGAATTAGCGAAC 2789
C35050   AAACTGCTAACCTTATTCATGGTATTTCAGACAGGATGCTTTGGTTTCTGTGGTAGACAAGTGGATCAAATTGACTACTACCGGGCTAGAATTAGCGAAC 2800
SN483    AAACTGCTAACCTTATTCATGGTATTTCAGACAGGATGCTTTGGTTTCTGTGGTAGACAAGTGGATCAAATTGACTACTACCGGGCTAGAATTAGCGAAC 2789

CS       TTGATAAGAGGGTACGCCTACAATTCGTTTGTGTCATGATTCCTTATATTTGAGAGGGAAAAAAAATTACCGTGCACGTTGAATTTCTACACCCCATGCA 2889
C35050   TTGATAAGAGGGTACGTCTACAATTCGTTTGTGTCATGATTCCTTATATTTGAGAGGGAAAAAAA.TTACCGTGCACGTTGAATTTCTACACCCCATGCA 2899
SN483    TTGATAAGAGGGTACGCCTACAATTCGTTTGTGTCATGATTCCTTATATTTGAGAGGGAAAAAAAATTACCGTGCACGTTGAATTTCTACACCCCATGCA 2889

CS       CATGTACCTATGTATATGATTTTAACATACATTTACGTTGATATTCTCAGATTGCATCGGAGCGTCACAGAGTTCTGAATGATCCAAAAGCCGTTATGCC 2989
C35050   CATGTACCTATGTATATGATTTTAACATACATTTACGTTGATATTCTCAGATTGCATCGGAGCGTCACAGAGTTCTGAATGATCCAAAAGCCGTTATGCC 2999
SN483    CATGTACCTATGTATATGATTTTAACATACATTTACGTTGATATTCTCAGATTGCATCGGAGCGTCACAGAGTTCTGAATGATCCAAAAGCCGTTATGCC 2989

CS       AGTTTCTTTTGTGACATTTGACTCAAGATGGGGTGCTGCTGTATGTGCACAGACACAACAGTCAAAGAATCCCACACAATGGCTGACCAATTGGGCTCCT 3089
C35050   AGTTTCTTTTGTGACATTTGACTCAAGATGGGGTGCTGCTGTATGTGCACAGACACAACAGTCAAAGAATCCCACACAATGGCTGACCAATTGGGCTCCT 3099
SN483    AGTTTCTTTTGTGACATTTGACTCAAGATGGGGTGCTGCTGTATGTGCACAGACACAACAGTCAAAGAATCCCACACAATGGCTGACCAATTGGGCTCCT 3089

CS       GAACCACGCGACGTTTATTGGCAGAATCTTGCTATTCCATTTTTCTCTCTGAGCATACGCAAGTTTCTCATATCAATCGCGGTCTTTGCTTTGGTGTTCT 3189
C35050   GAACCACGCGACGTTTATTGGCAGAATCTTGCTATTCCATTTTTCTCTCTGAGCATACGCAAGTTTCTCATATCAATCGCGGTCTTTGCTTTGGTGTTCT 3199
SN483    GAACCACGCGACGTTTATTGGCAGAATCTTGCTATTCCATTTTTCTCTCTGAGCATACGCAAGTTTCTCATATCAATCGCGGTCTTTGCTTTGGTGTTCT 3189

CS       TCTACATGATACCTATTGCATTTGTGCAATCACTTGCCAATCTCGAGGGTATTGAAAGAGTTGCGCCTTTCTTGAGGCCAGTAATAGAAGTGTAAGAATT 3289
C35050   TCTACATGATACCTATTGCATTTGTGCAATCACTTGCCAATCTCGAGGGTATTGAAAGAGTTGCGCCTTTCTTGAGGCCAGTAATAGAAGTGTAAGAATT 3299
SN483    TCTACATGATACCTATTGCATTTGTGCAATCACTTGCCAATCTCGAGGGTATTGAAAGAGTTGCGCCTTTCTTGAGGCCAGTAATAGAAGTGTAAGAATT 3289

CS       TTTTTTT.CTTTTTGCACATCATGCTTCTTGAAATTGCTCATTATTTTTGTTCATTCACACTGACTTATTTCAACTTGCAGAAAAGTGGTGAAGTCTTTC 3388
C35050   TTTTTTTTCTTTTTGCACATCATGCTTCTTGAAATTGCTCATTATTTTTGTTCATTCACACTGACTTATTTCAACTTGCAGAAAAGTGGTGAAGTCTTTC 3399
SN483    TTTTTTT.CTTTTTGCACATCATGCTTCTTGAAATTGCTCATTATTTTTGTTCATTCACACTGACTTATTTCAACTTGCAGAAAAGTGGTGAAGTCTTTC 3388
```

Supplementary Figure 2. Continued

```
CS       CTGCAGGGTTTCCTACCCGGTCTGGCTTTGAAGCTCTTTCTCTATATCCTCCCTACGGTTCTTATGATCATGTCAAAAGTTGAAGGCTATGTCTCTTTGT   3488
C35050   CTGCAGGGTTTCCTACCCGGTCTGGCTTTGAAGCTCTTTCTCTATATCCTCCCTACGGTTCTTATGATCATGTCAAAAGTTGAAGGCTATGTCTCTTTGT   3499
SN483    CTGCAGGGTTTCCTACCCGGTCTGGCTTTGAAGCTCTTTCTCTATATCCTCCCTACGGTTCTTATGATCATGTCAAAAGTTGAAGGCTATGTCTCTTTGT   3488

CS       CATCGCTGGAAAGAAGAACTGCTTCGAAATATTACTACTTCATGCTGGTGAATGTATTTCTTGGAAGCATAATTGCTGGCACAGCTTTTGAACAGCTAGA   3588
C35050   CATCGCTGGAAAGAAGAACTGCTTCGAAATATTACTACTTCATGCTGGTGAATGTATTTCTTGGAAGCATAATTGCTGGCACAGCTTTTGAACAGCTAGA   3599
SN483    CATCGCTGGAAAGAAGAACTGCTTCGAAATATTACTACTTCATGCTGGTGAATGTATTTCTTGGAAGCATAATTGCTGGCACAGCTTTTGAACAGCTAGA   3588

CS       CTCTTTTTTTCACGATCCTCCTTCACAGTAAGTTTCTTCAAACATTCTTCTGCCAACATGCATACATGAACCAAAACTTATTTTCCCATGTCTTCATTAC   3688
C35050   CTCTTTTTTTCACGATCCTCCTTCACAGTAAGTTTCTTCAAACATTCTTCTGCCAACATGCATACATGAACCAAAACTTATTTTCCCATGTCTTCATTAC   3699
SN483    CTCTTTTTTTCACGATCCTCCTTCACAGTAAGTTTCTTCAAACATTCTTCTGCCAACATGCATACATGAACCAAAACTTATTTTCCCATGTCTTCATTAC   3688

CS       CTTAATTATCCCCTTTTCGGAAAGAAAAATGTCTAAATTTCATTAATCATTGTGCCGCCGCTTCATATGGCTAGAATGTACTAGTACTCACTCTCAAAAT   3788
C35050   CTTAATTATCCCCTTTTCGGAAAGAAAAATGTCTAAATTTCATTAATCATTGTGCCGCCGCTTCATATGGCTAGAATGTACTAGTACTCACTCTCAAAAT   3799
SN483    CTTAATTATCCCCTTTTCGGAAAGAAAAATGTCTAAATTTCATTAATCATTGTGCCGCCGCTTCATATGGCTAGAATGTACTAGTACTCACTCTCAAAAT   3788

CS       GTACAAACGAAAAATGTTGGATGCATAGGCATAAACAGTAACAATCTGTCACGTCTCCTTTTTTTTCTTTACAGAATACCAAGGACCATTGGAGTGGCTG   3888
C35050   GTACAAACGAAAAATGTTGGATGCATAGGCATAAACAGTAACAATCTGTCACGTCTCCTCTTTTTTCTTTACAGAATACCAAGGACCATTGGAGTGGCTG   3899
SN483    GTACAAACGAAAAATGTTGGATGCATAGGCATAAACAGTAACAATCTGTCACGTCTCCTTTTTTTTCTTTACAGAATACCAAGGACCATTGGAGTGGCTG   3888

CS       TACCAATGAAAGCAACATTCTTCATGACATACATAATGGTCGATGGCTGGGCCGGCATCGCCAATGAGATTCTTCGAGTGAAGCCACTGGTCATATACCA   3988
C35050   TACCAATGAAAGCAACATTCTTCATGACATACATAATGGTCGATGGCTGGGCCGGCATCGCCAATGAGATTCTTCGAGTGAAGCCACTGGTCATATACCA   3999
SN483    TACCAATGAAAGCAACATTCTTCATGACATACATAATGGTCGATGGCTGGGCCGGCATCGCCAATGAGATTCTTCGAGTGAAGCCACTGGTCATATACCA   3988

CS       TCTGAAGAACATGTTCATCGTGAAAACCGAGCGGGACAGGGAGAGGGCGATGGATCCACGGAGCATCGCGCTCGGAGAAAACCTTCCATCCCTGCAGCTG   4088
C35050   TCTGAAGAACATGTTCATCGTGAAAACCGAGCGGGACAGGGAGAGGGCGATGGATCCACGGAGCATCGCGCTCGGAGAAAACCTTCCATCCCTGCAGCTG   4099
SN483    TCTGAAGAACATGTTCATCGTGAAAACCGAGCGGGACAGGGAGAGGGCGATGGATCCACGGAGCATCGCGCTCGGAGAAAACCTTCCATCCCTGCAGCTG   4088

CS       TACTTCCTCCTTGGGCTCGTGTACGCGGTGGTCACCCCCATTCTCCTTCCTTTCATAATCGTCTTCTTCGCCTTCGCCTACCTTGTCTACAGGCATCAGG   4188
C35050   TACTTCCTCCTTGGGCTCGTGTACGCGGTGGTCACCCCCATTCTCCTTCCTTTCATAATCGTCTTCTTCGCCTTCGCCTACCTTGTCTACAGGCATCAGG   4199
SN483    TACTTCCTCCTTGGGCTCGTGTACGCGGTGGTCACCCCCATTCTCCTTCCTTTCATAATCGTCTTCTTCGCCTTCGCCTACCTTGTCTACAGGCATCAGG   4188

CS       TGAGTTGATGAATACTGCTTTGACCTCTAGCCATTTGCTGTGTCAAAGACAGTGCACTGACATACCTCCATTCCTGGTTTCAGATCATCAATGTGTACAA   4288
C35050   TGAGTTGATGAATACTGCTTTGACCTCTAGCCATTTGCTGTGTCAAAGACAGTGCCCTGACATACCTCCATTCCTGGTTTCAGATCATCAATGTGTACAA   4299
SN483    TGAGTTGATGAATACTGCTTTGACCTCTAGCCATTTGCTGTGTCAAAGACAGTGCACTGACATACCTCCATTCCTGGTTTCAGATCATCAATGTGTACAA   4288

CS       CCAGGAGTATGAGAGCGCGGCGGCATTCTGGCCTCAGGTCCATTCGCGCATCATAGCGAGCCTGCTCATCTCCCATGTGACTCTCTTTGGGCTGCTGAGC   4388
C35050   CCAGGAGTATGAGAGCGCGGCGGCATTCTGGCCTCAGGTCCATTCGCGCATCATAGCGAGCCTGCTCATCTCCCATGTGACTCTCTTTGGGCTGCTGAGC   4399
SN483    CCAGGAGTATGAGAGCGCGGCGGCATTCTGGCCTCAGGTCCATTCGCGCATCATAGCGAGCCTGCTCATCTCCCATGTGACTCTCTTTGGGCTGCTGAGC   4388

CS       ACGATGAAAGCCGCCTACTCCACCCCGCTGCTCATCTTCCTGCCGTTGCTGACACTATGGTTCCACAAGTACTGCAAGAGTCGGTTCGAGCCCGCTTTCC   4488
C35050   ACGATGAAAGCCGCCTACTCCACCCCGCTGCTCATCTTCCTGCCGTTGCTGACACTATGGTTCCACAAGTACTGCAAGAGTCGGTTCGAGCCCGCTTTCC   4499
SN483    ACGATGAAAGCCGCCTACTCCACCCCGCTGCTCATCTTCCTGCCGTTGCTGACACTATGGTTCCACAAGTACTGCAAGAGTCGGTTCGAGCCCGCTTTCC   4488

CS       GCAAGTATCCTCTAGAGGTCACTGCTTCGCCTCTGTCTGTGTCACGTGTGATTTTTGCTTGGATGATCGTTTGCAAGACCTGAAATGTTGTTTGCTTTTC   4588
C35050   GCAAGTATCCTCTAGAGGTCACTGCTTCGCCTCTGTCTGTGTCACGTGTGATTTTTGCTTGGATGATCGTTTGCAAGACCTGAAATGTTGTTTGCTTTTC   4599
SN483    GCAAGTATCCTCTAGAGGTCACTGCTTCGCCTCTGTCTGTGTCACGTGTGATTTTTGCTTGGATGATCGTTTGCAAGACCTGAAATGTTGTTTGCTTTTC   4588

CS       TGAACTTCAGGAAGCGATGGAGAAGGACGTGATGGAGCACGCGTCGGAGCCGAGCCTGAACCTCAAGACCTACCTGGCAAATGCTTACCTGCACCCCATC   4688
C35050   TGAACTTCAGGAAGCGATGGAGAAGGACGTGATGGAGCACGCGTCGGAGCCGAGCCTGAACCTCAAGACCTACCTGGCAAATGCTTACCTGCACCCCATC   4699
SN483    TGAACTTCAGGAAGCGATGGAGAAGGACGTGATGGAGCACGCGTCGGAGCCGAGCCTGAACCTCAAGACCTACCTGGCAAATGCTTACCTGCACCCCATC   4688

CS       TTCCATATGTTTGAGCAGGAGGATCAGAAAGAGGAGGCCACCATAGAGGTGAGAATCGACAAATCGGAGCAGCAGCAGC......AGCATGTGAGAAGCT   4782
C35050   TTCCATATGTTTGAGCAGGAGGATCAGAAAGAGGAGGCCACCATAGAGGTGAGAATCGACAAATCGGAGCAGCAGCAGCAGCAGCAGCATGTGAGAAGCT   4799
SN483    TTCCATATGTTTGAGCAGGAGGATCAGAAAGAGGAGGCCACCATAGAGGTGAGAATCGACAAATCGGAGCAGCAGCAGC......AGCATGTGAGAAGCT   4782

CS       CACACTCACAGTATGAGGAGGAGACGAGCGCACAGACGCATTACCACCATGAGGAGAGGAGCAGCAGCCAGTACCAGTACCAGTACCAGCACCAGTATCA   4882
C35050   CACACTCACAGTATGAGGAGGAGACGAGCGCACAGACGCATTACCACCATGAGGAGAGGAGCAGCAGCCGGTACCAGTACCAGTACCAGCACCAGTATCA   4899
SN483    CACACTCACAGTATGAGGAGGAGACGAGCGCACAGACGCATTACCACCATGAGGAGAGGAGCAGCAGCCAGTACCAGTACCAGTACCAGCACCAGTATCA   4882

CS       GTACCAGCACGAGGAAACTCACATGAGGAGTGAGCAGTCGCCGCCACACTTTGTCTACCACCATGGAGTCGAGCACTGA                        4961
C35050   GTACCAGCACGAGGAAACTCACATGAGGAGTGAGCAGTCGCCGCCACACTTTGTCTACCACCATGGAGTCGAGCACTGA                        4978
SN483    GTACCAGCACGAGGAAACTCACATGAGGAGTGAGCAGTCGCCGCCACACTTTGTCTACCACCATGGAGTCGAGCACTGA                        4961
```

Supplementary Figure 2. Continued
