# Supplementary material for: Cloning a novel reduced-height (Rht) gene TaOSCA1.4 from a QTL in wheat
Source: Front Plant Sci. 2024 May 16;15:1381243. doi: 10.3389/fpls.2024.1381243 (PMC11137288; doi:10.3389/fpls.2024.1381243)
Supplement: Supplementary file 6 [file DataSheet_3.pdf]

```
CS       MATLQDLGVSAFINILGAFVFLLLFAVLRIQPINDRVYFPKLYIAGKRAADHRGARRAINLNLCTYFKFLSWVPGALRMTQTELIHHAGLDSAVYLRIYT  100
C35050   MATLQDLGVSAFINILGAFVFLLLFAVLRIQPINDRVYFPKLYIAGKRAADHRGARRAINLNLCTYFKFLSWVPGALRMTQTELIHHAGLDSAVYLRIYT  100
SN483    MATLQDLGVSAFINILGAFVFLLLFAVLRIQPINDRVYFPKLYIAGKRAADHRGARRAINLNLCTYFKFLSWVPGALRMTQTELIHHAGLDSAVYLRIYT  100

CS       LGLKIFLPVMVVALLVLIPVNVSGGTLLNLRKDVMFSDIDKLSISNVSPGSNRFFIHLLMAYVFTFWTCFMLYKEYSNVAFMRLHFLASQKRCADQFTVI  200
C35050   LGLKIFLPVMVVALLVLIPVNVSGGTLLNLRKDVMFSDIDKLSISNVSPGSNRFFIHLLMAYVFTFWTCFMLYKEYSNVAFMRLHFLASQKRCADQFTVI  200
SN483    LGLKIFLPVMVVALLVLIPVNVSGGTLLNLRKDVMFSDIDKLSISNVSPGSNRFFIHLLMAYVFTFWTCFMLYKEYSNVAFMRLHFLASQKRCADQFTVI  200

CS       VRNIPRVSSHSTSEAVDEFFRRNHPDHYLGQQAVYNANRYAKLVKQKERLQNWLDYYQLKFERHPEKRPTGRTGCFGFCGRQVDQIDYYRARISELDKRI  300
C35050   VRNIPRVSSHSTSEAVDEFFRRNHPDHYLGQQAVYNANRYAKLVKQKERLQNWLDYYQLKFERHPEKRPTGRTGCFGFCGRQVDQIDYYRARISELDKRI  300
SN483    VRNIPRVSSHSTSEAVDEFFRRNHPDHYLGQQAVYNANRYAKLVKQKERLQNWLDYYQLKFERHPEKRPTGRTGCFGFCGRQVDQIDYYRARISELDKRI  300

CS       ASERHRVLNDPKAVMPVSFVTFDSRWGAAVCAQTQQSKNPTQWLTNWAPEPRDVYWQNLAIPFFSLSIRKFLISIAVFALVFFYMIPIAFVQSLANLEGI  400
C35050   ASERHRVLNDPKAVMPVSFVTFDSRWGAAVCAQTQQSKNPTQWLTNWAPEPRDVYWQNLAIPFFSLSIRKFLISIAVFALVFFYMIPIAFVQSLANLEGI  400
SN483    ASERHRVLNDPKAVMPVSFVTFDSRWGAAVCAQTQQSKNPTQWLTNWAPEPRDVYWQNLAIPFFSLSIRKFLISIAVFALVFFYMIPIAFVQSLANLEGI  400

CS       ERVAPFLRPVIEVKVVKSFLQGFLPGLALKLFLYILPTVLMIMSKVEGYVSLSSLERRTASKYYYFMLVNVFLGSIIAGTAFEQLDSFFHDPPSQIPRTI  500
C35050   ERVAPFLRPVIEVKVVKSFLQGFLPGLALKLFLYILPTVLMIMSKVEGYVSLSSLERRTASKYYYFMLVNVFLGSIIAGTAFEQLDSFFHDPPSQIPRTI  500
SN483    ERVAPFLRPVIEVKVVKSFLQGFLPGLALKLFLYILPTVLMIMSKVEGYVSLSSLERRTASKYYYFMLVNVFLGSIIAGTAFEQLDSFFHDPPSQIPRTI  500

CS       GVAVPMKATFFMTYIMVDGWAGIANEILRVKPLVIYHLKNMFIVKTERDRERAMDPRSIALGENLPSLQLYFLLGLVYAVVTPILLPFIIVFFAFAYLVY  600
C35050   GVAVPMKATFFMTYIMVDGWAGIANEILRVKPLVIYHLKNMFIVKTERDRERAMDPRSIALGENLPSLQLYFLLGLVYAVVTPILLPFIIVFFAFAYLVY  600
SN483    GVAVPMKATFFMTYIMVDGWAGIANEILRVKPLVIYHLKNMFIVKTERDRERAMDPRSIALGENLPSLQLYFLLGLVYAVVTPILLPFIIVFFAFAYLVY  600

CS       RHQIINVYNQEYESAAAFWPQVHSRIIASLLISHVTLFGLLSTMKAAYSTPLLIFLPLLTLWFHKYCKSRFEPAFRKYPLEEAMEKDVMEHASEPSLNLK  700
C35050   RHQIINVYNQEYESAAAFWPQVHSRIIASLLISHVTLFGLLSTMKAAYSTPLLIFLPLLTLWFHKYCKSRFEPAFRKYPLEEAMEKDVMEHASEPSLNLK  700
SN483    RHQIINVYNQEYESAAAFWPQVHSRIIASLLISHVTLFGLLSTMKAAYSTPLLIFLPLLTLWFHKYCKSRFEPAFRKYPLEEAMEKDVMEHASEPSLNLK  700

CS       TYLANAYLHPIFHMFEQEDQKEEATIEVRIDKSEQQQQ..HVRSSHSQYEEETSAQTHYHHEERSSSQYQYQYQHQYQYQHEETHMRSEQSPPHFVYHHG  798
C35050   TYLANAYLHPIFHMFEQEDQKEEATIEVRIDKSEQQQQQQHVRSSHSQYEEETSAQTHYHHEERSSSRYQYQYQHQYQYQHEETHMRSEQSPPHFVYHHG  800
SN483    TYLANAYLHPIFHMFEQEDQKEEATIEVRIDKSEQQQQ..HVRSSHSQYEEETSAQTHYHHEERSSSQYQYQYQHQYQYQHEETHMRSEQSPPHFVYHHG  798

CS       VEH  801
C35050   VEH  803
SN483    VEH  801
```

Supplementary Figure 3. Difference of amino acid sequences between C35050 and SN483 for *TaOSCA1.4* gene.
